# Evaluation of Oxford nanopore sequencing for antimicrobial resistance surveillance in *Salmonella*: comparison with phenotypic antimicrobial susceptibility in a large-scale study

**DOI:** 10.1128/spectrum.01797-26

**Published:** 2026-08-12

**Authors:** Yu-Ping Hong, Ying-Shu Liao, You-Wun Wang, Shao-Chun Kuo, Ru-Hsiou Teng, Shiu-Yun Liang, Jui-Hsien Chang, Hsiao Lun Wei, Chien-Shun Chiou

**Affiliations:** 1 Center for Research, Diagnostics and Vaccine Development, Centers for Disease Control, Ministry of Health and Welfare, Taichung, Taiwan; Instituto Nacional de Tecnologia Agropecuaria, Buenos Aires, Argentina

**Keywords:** non-typhoidal *Salmonella *(NTS), antimicrobial susceptibility testing (AST), antimicrobial resistance (AMR), whole-genome sequencing (WGS), Oxford nanopore sequencing

## Abstract

*Salmonella* is a major zoonotic foodborne pathogen, and antimicrobial resistance (AMR) in *Salmonella* presents a significant public health challenge. Compared with conventional antimicrobial susceptibility testing (AST), whole-genome sequencing (WGS) provides a more rapid and comprehensive approach to AMR characterization, thereby informing antimicrobial selection and supporting public health surveillance. In this study, Oxford Nanopore Technology (ONT)-based WGS was performed on 1,490 *Salmonella* isolates collected through nationwide surveillance in Taiwan in 2025. Genotypic resistance inferred from WGS data was compared with phenotypic AST results to assess the performance of ONT-WGS. Overall, WGS-inferred resistance showed high concordance with phenotypic resistance for most antimicrobials. However, major genotype–phenotype discordance was observed, attributed to four categories: (i) breakpoint-dependent classification, (ii) reduced or absent phenotypic expression of resistance genes, (iii) minimum inhibitory concentration (MIC) modulation by *ramAp*, and (iv) absence of known AMR determinants. Notable discrepancies included tigecycline resistance without known genetic determinants, nalidixic acid resistance linked to *ramAp*-mediated MIC elevation, and a high prevalence of colistin resistance (35.7%) in *S.* Enteritidis, with most resistant isolates lacking identifiable AMR determinants. Additionally, a significant proportion of ESBL- and AmpC-producing isolates were classified as susceptible or intermediate to cefotaxime and ceftazidime under CLSI criteria, highlighting the potential for misclassification and treatment failure. These findings demonstrate that ONT-WGS enables accurate and comprehensive AMR characterization by directly identifying resistance determinants and avoiding potential misclassification associated with breakpoint-based AST interpretations. When interpreted appropriately, WGS can support better antimicrobial selection and serve as a valuable alternative to conventional susceptibility testing.

**IMPORTANCE:**

Accurate prediction of antimicrobial resistance is essential for appropriate therapy and effective surveillance of *Salmonella*. However, discordance between genotype-based predictions and phenotypic antimicrobial susceptibility testing (AST) can complicate clinical interpretation. In this nationwide study of 1,490 Salmonella isolates, we show that Oxford Nanopore Technology-based whole-genome sequencing (ONT-WGS) provides rapid and comprehensive detection of antimicrobial resistance determinants with high concordance to phenotypic AST. We further identify four major mechanisms underlying genotype–phenotype discordance, including breakpoint-dependent classification, reduced or absent phenotypic expression of resistance genes, minimum inhibitory concentration (MIC) modulation by *ramAp*, and the absence of known AMR determinants. These findings demonstrate how WGS can complement conventional AST, improve interpretation of challenging susceptibility results, and strengthen genomic surveillance of emerging antimicrobial-resistant *Salmonella*.

## INTRODUCTION

*Salmonella* is a leading zoonotic foodborne pathogen globally, responsible for millions of cases of gastroenteritis each year. Antimicrobial resistance (AMR) in *Salmonella* has become a critical issue, as it diminishes the effectiveness of treatment options, leading to increased morbidity, mortality, and healthcare costs (1, 2). The World Health Organization has designated fluoroquinolone-resistant *Salmonella* as a high-priority pathogen requiring urgent surveillance and control (3). In Taiwan, long-term surveillance has revealed a steady rise in *Salmonella* resistance to key antimicrobials (4). Over the past decade, high-risk multidrug-resistant (MDR) *Salmonella* clones have repeatedly emerged, causing large-scale outbreaks. Notable examples include MDR *S*. Anatum, azithromycin-resistant extensively drug-resistant (AziR-XDR) *S*. Typhimurium, MDR *S*. Goldcoast, MDR *S*. Agona, MDR *S*. Infantis, and, more recently, AziR-XDR *S*. Kentucky (5–10).

Antimicrobial susceptibility testing (AST) has long been the gold standard for determining phenotypic resistance. However, its utility is limited by lengthy turnaround times and the restricted range of antimicrobials routinely tested. Although AST can detect phenotypic resistance even in the absence of known AMR determinants, isolates carrying resistance genes may still be classified as susceptible when their MICs fall below clinical breakpoints, potentially increasing the risk of treatment failure. In contrast, whole-genome sequencing (WGS) has become a more efficient and comprehensive alternative, as it enables the direct identification of AMR determinants, precise serotype prediction, high-resolution genotyping, and source attribution (11–14). Unlike AST, WGS can detect all known AMR determinants, including those linked to drugs not included in the AST panels.

Illumina and Oxford Nanopore Technologies (ONT) are the two most widely used platforms for WGS of bacterial isolates. Illumina short-read sequencing is well established for its high per-base accuracy and robust performance in AMR detection and genomic epidemiology. Previous large-scale studies have demonstrated that Illumina-based WGS can accurately predict phenotypic antimicrobial resistance in *Salmonella*, establishing the feasibility of genome-based antimicrobial resistance prediction for routine surveillance (15, 16). However, its long turnaround time and limited operational flexibility, including the need for centralized laboratory infrastructure and batch sequencing, limit its utility for real-time AMR detection and disease surveillance. Because Illumina sequencing is most cost-effective when large numbers of isolates are processed together, individual or low-volume samples often experience delays while awaiting the completion of the batch. In contrast, ONT long-read sequencing offers faster turnaround times, greater operational flexibility, and cost-effectiveness for sequencing smaller numbers of isolates, making it more adaptable for bacterial WGS. While earlier ONT platforms faced challenges with basecalling errors (17), recent advances in nanopore chemistry and basecalling algorithms have significantly improved accuracy, with studies showing comparable performance to Illumina for core-genome multilocus sequence typing (cgMLST) and single-nucleotide polymorphism (SNP)-based analyses (13, 18). These improvements make ONT a practical platform for real-time genomic surveillance of bacterial pathogens. ONT has already been successfully used to identify *Salmonella* serotypes and AMR determinants (19). Accordingly, the remaining challenge is no longer whether WGS can accurately predict antimicrobial resistance, but whether the more rapid and operationally flexible ONT platform can deliver performance comparable to Illumina’s for routine large-scale genomic surveillance. However, comparable large-scale evaluations using Oxford Nanopore sequencing remain limited. Notably, the concordance between genotypic AMR profiles and phenotypic AST results has not been systematically assessed using large collections of *Salmonella* isolates.

In this study, we applied ONT sequencing to a large-scale collection of 1,490 *Salmonella* isolates obtained through nationwide surveillance in Taiwan in 2025. We conducted comprehensive genomic characterization, including the identification of AMR determinants, serotype prediction, multilocus sequence typing (MLST), and plasmid replicon profiling. The primary objective was to evaluate the performance of ONT-based WGS for predicting AMR. Specifically, we assessed the concordance between genotypic AMR profiles and phenotypic AST results. Additionally, we utilized WGS data to track the circulation of major MDR and XDR *Salmonella* clones previously reported in Taiwan.

## MATERIALS AND METHODS

### Bacterial isolates

A total of 1,490 *Salmonella* isolates were collected from hospitals across Taiwan through a nationwide surveillance program. Clinical isolates submitted by participating hospitals were initially cultured on Salmonella–Shigella (SS) agar for selective isolation and purification. Presumptive *Salmonella* colonies were identified based on characteristic colony morphology and subsequently subcultured onto tryptic soy agar (TSA) to obtain sufficient biomass for downstream applications. Genomic DNA was extracted from TSA-grown cultures for WGS. In parallel, purified isolates grown on TSA were preserved for long-term storage in tryptic soy broth supplemented with 15% glycerol at −80°C. For AST, TSA-grown isolates were subcultured onto blood agar plates to ensure optimal growth prior to testing.

### Whole-genome sequencing and analysis

ONT-WGS was performed using the MinION devices (Mk1B or Mk1D) from Oxford Nanopore Technologies (Oxford, United Kingdom). Genomic DNA was extracted from TSA-grown cultures using the EZ2 PowerFecal Pro DNA/RNA Kit (cat. no. 954634; Qiagen, Hilden, Germany), according to the manufacturer’s instructions. Sequencing libraries were prepared using the Rapid Barcoding Kit (SQK-RBK114; Oxford Nanopore Technologies) and sequenced on MinION devices equipped with R10.4.1 flow cells (FLO-MIN114). Raw signal data (POD5 format) were basecalled using Dorado v1.1.1 (Oxford Nanopore Technologies) with the Super Accurate (SUP5.2.0) model to generate FASTQ reads. Long-read assemblies were generated using Flye v2.9.6 (20). Circular contigs were re-oriented using Dnaapler v1.2.0 (21) and subsequently polished using Medaka v2.0.1 (Oxford Nanopore Technologies). Raw ONT data were accepted only when sequencing depth reached ≥20× , which had previously been established in our laboratory as sufficient for reliable identification of AMR determinants, including β-lactamase subtypes. Following assembly polishing with Medaka, all ONT data sets were evaluated using Alpaqa (18), which analyzes consensus per-base quality scores to identify assemblies potentially affected by systematic basecalling errors associated with DNA modifications or sample contamination. Isolates flagged with abnormal quality metrics were re-sequenced before downstream analyses.

*In silico* serotype prediction was performed using SISTR v1.1.3 (22), and sequence types (STs) were assigned using mlst v2.32.2 (https://github.com/tseemann/mlst). Plasmid replicon types were identified using PlasmidFinder v2.2.0 (23). AMR determinants, including acquired resistance genes and resistance-associated mutations, were identified using AMRFinderPlus v4.0.3 (24).

### Antimicrobial susceptibility testing

AST was performed using the EUVSEC3 Sensititre broth microdilution MIC panel (TREK Diagnostic Systems Ltd., Thermo Fisher Scientific, East Grinstead, United Kingdom), which includes 15 antimicrobial agents recommended for antimicrobial resistance monitoring in *Salmonella* under the European Union surveillance protocol. Minimum inhibitory concentrations (MICs) were interpreted according to the Clinical and Laboratory Standards Institute (CLSI) guidelines (25) for 14 antimicrobial agents. For tigecycline, interpretive criteria were based on the European Committee on Antimicrobial Susceptibility Testing (EUCAST) recommendations (26). MDR isolates were defined as isolates resistant to the antimicrobial agents traditionally used to treat *Salmonella* infections, including ampicillin, chloramphenicol, and trimethoprim-sulfamethoxazole. XDR isolates were defined as MDR isolates that additionally exhibited nonsusceptibility to fluoroquinolones and resistance to third-generation cephalosporins. AziR-XDR isolates were defined as XDR isolates that were also resistant to azithromycin. In cases where phenotypic resistance and AMR determinant-inferred resistance were inconsistent for one or more antimicrobial agents, AST or WGS was repeated to verify.

## RESULTS

### Phenotypic and genotypic resistance

Among the 1,490 *Salmonella* isolates, phenotypic AMR varied across antimicrobial classes (Table 1). High resistance rates (>50%) were observed for ampicillin (60.5%), sulfamethoxazole (60.8%), tetracycline (60.3%), and chloramphenicol (53.2%). A substantial proportion of isolates also exhibited ciprofloxacin nonsusceptibility (46.4%) and trimethoprim resistance (45.2%). Moderate resistance rates were noted for cefotaxime (21.5%), tigecycline (20.1%), ceftazidime (19.3%), nalidixic acid (14.8%), and gentamicin (11.7%), whereas a lower resistance rate was observed for azithromycin (4.4%). Resistance to amikacin and meropenem was rare, each detected in a single isolate (0.1%). For colistin, resistance differed markedly by serovar. Colistin resistance was rare (0.7%) in non-*S*. Enteritidis isolates, whereas a high resistance rate (35.7%) was observed in *S.* Enteritidis isolates.

**TABLE 1:** Comparison of antimicrobial resistance determined by phenotypic AST and inferred from WGS-identified resistance determinants among 1,490 *Salmonella* isolates^*a*^^,*b*^

Antimicrobial	Phenotypic resistance		Genotypic resistance		Resistance determinants
	No. isolates	Resistance, %	No. isolates (included *ramAp*)	Resistance, % (included *ramAp*)	
Amikacin	1	0.1	1	0.1	*rmtB1*
Ampicillin	902	60.5	905	60.7	*ampC, bla*_CMY_ *, bla*_CMY-2_ *, bla*_CTX-M_ *, bla*_CTX-M-14_ *, bla*_CTX-M-15_ *, bla*_CTX-M-3_ *, bla*_CTX-M-55_ *, bla*_CTX-M-65_ *, bla*_DHA-1_ *, bla*_KPC-2_ *, bla*_LAP-2_ *, bla*_OXA_ *, bla*_OXA-10_ *, bla*_PSE_ *, bla*_TEM_ *, bla*_TEM-1_ *, bla*_TEM-135_ *, bla*_TEM-176_
Azithromycin	66	4.4	61 (196)	4.1 (13.2)	*erm(42), erm(B), mph(A), ramAp*
Cefotaxime	320	21.5	351	23.6	*ampC, bla*_CMY_ *, bla*_CMY-2_ *, bla*_CTX-M_ *, bla*_CTX-M-14_ *, bla*_CTX-M-15_ *, bla*_CTX-M-3_ *, bla*_CTX-M-55_ *, bla*_CTX-M-65_ *, bla*_DHA-1_ *, bla*_KPC-2_
Ceftazidime	288	19.3	351	23.6	*ampC, bla*_CMY_ *, bla*_CMY-2_ *, bla*_CTX-M_ *, bla*_CTX-M-14_ *, bla*_CTX-M-15_ *, bla*_CTX-M-3_ *, bla*_CTX-M-55_ *, bla*_CTX-M-65_ *, bla*_DHA-1_ *, bla*_KPC-2_
Chloramphenicol	793	53.2	789 (795)	53.0 (53.3)	*catA1, catA2, cmlA1, cmlA5, floR, ramAp*
Ciprofloxacin^(NS)^	692	46.4	696 (710)	46.7 (47.7)	*aac(6')-Ib-cr, gyrA_D87G, gyrA_D87N, gyrA_D87Y, gyrA_S83F, gyrA_S83Y, parC_S80I, qepA8, qnrA1, qnrB, qnrB6, qnrD1, qnrS, qnrS1, qnrS13, ramAp*
Colistin (non-*S*. Enteritidis, *N* = 1,232)	9	0.7	9	0.7	*mcr-1.1*
Colistin (*S*. Enteritidis, *N* = 258)	92	35.7	1	0.4	*mcr-1.1*
Gentamicin	174	11.7	174	11.7	*aac(3)-IId, aac(3)-IIe, aac(3)-IVa, rmtB1*
Meropenem	1	0.1	1	0.1	*bla*_KPC-2_
Nalidixic acid	221	14.8	118 (253)	7.9 (17.0)	*gyrA_D87G, gyrA_D87N, gyrA_D87Y, gyrA_S83F, gyrA_S83Y, parC_S80I, ramAp*
Sulfamethoxazole	906	60.8	907 (914)	60.9 (61.3)	*ramAp, sul1, sul2, sul3*
Tetracycline	899	60.3	904 (907)	60.7 (60.9)	*ramAp, tet(A), tet(B), tet(D), tet(G), tet(M*)
Tigecycline	299	20.1	0 (138)	0 (9.3)	*ramAp*
Trimethoprim	673	45.2	674 (677)	45.2 (45.4)	*dfrA1, dfrA12, dfrA14, dfrA17, dfrA23, dfrA25, dfrA27, dfrA5, dfrA51, dfrA7, ramAp*
Clindamycin	NT	NT	322	21.6	*erm(42), erm(B), lnu(F*)
Erythromycin	NT	NT	93	6.2	*erm(42), erm(B), estT, mph(A*)
Fosfomycin	NT	NT	203	13.6	*fosA3, fosA7, fosA7.2, fosA7.3, fosA7.4*
Hygromycin B	NT	NT	31	2.1	*aph(4)-Ia*
Kanamycin	NT	NT	333	22.3	*aac(6'), aac(6')-Ib-cr, aph(3')-Ia, aph(3')-Iia*
Rifampin	NT	NT	166	11.1	*arr-2, arr-3*
Streptomycin	NT	NT	896	60.1	*aadA1, aadA16, aadA2, aadA22, aadA7, aph(3'')-Ib, aph(6)-Ic, aph(6)-Id*

^*a*^ Genotypic resistance was inferred from acquired resistance genes and resistance-associated mutations (AMR determinants).

^*b*^ Values in parentheses indicate the total number and percentage of isolates predicted to be resistant when *ramAp *was included as an AMR determinant. NS, non-susceptible; NT, not tested.

Genotypic resistance inferred from WGS-identified AMR determinants showed similar trends to those of phenotypic resistance for most antimicrobials (Table 1). Concordant or near-concordant resistance proportions (excluding the effect of *ramAp*) were observed for ampicillin (60.5% vs 60.7%), azithromycin (4.4% vs 4.1%), chloramphenicol (53.2% vs 53.0%), ciprofloxacin nonsusceptibility (46.4% vs 46.7%), gentamicin (11.7% vs 11.7%), meropenem (0.1% vs 0.1%), sulfamethoxazole (60.8% vs 60.9%), tetracycline (60.3% vs 60.7%), trimethoprim (45.2% vs 45.2%), and colistin in non-*S*. Enteritidis isolates (0.7% vs 0.7%).

Marked discrepancies between phenotypic and genotypic resistance were observed for selected antimicrobials. For tigecycline, no isolates were predicted to be resistant based on known AMR determinants, whereas 20.1% were phenotypically resistant according to the current EUCAST criteria (S ≤ 0.5; R > 0.5 mg/L). Similarly, for nalidixic acid, genotypic resistance (7.9%), excluding the effect of *ramAp,* was substantially lower than the observed phenotypic resistance (14.8%). In contrast, genotypic resistance exceeded phenotypic resistance for cefotaxime (23.6% vs 21.5%) and ceftazidime (23.6% vs 19.3%).

For colistin, a pronounced genotype–phenotype discrepancy was observed among *S.* Enteritidis isolates: 35.7% (92/258) were phenotypically resistant, whereas only one isolate (0.4%, 1/258) harbored the plasmid-mediated colistin resistance gene *mcr-1.1*. In contrast, among non-*S*. Enteritidis isolates, both phenotypic and genotypic resistance rates were low (0.7% and 0.7%, respectively). Consistent with this pattern, among isolates lacking known colistin resistance determinants, *S.* Enteritidis isolates exhibited a shift toward higher colistin MIC values, whereas non-*S*. Enteritidis isolates were predominantly distributed at lower MIC levels (Fig. S1).

Incorporation of *ramAp* as an AMR determinant markedly affected genotype-based resistance prediction for several antimicrobials (Table 1). *ramAp*, a plasmid-borne *ramA* homolog, has been shown to elevate expression of the AcrAB-TolC efflux system and to elevate MICs to multiple antimicrobials, including azithromycin, cefoxitin, chloramphenicol, ciprofloxacin, nalidixic acid, sulfamethoxazole, tetracycline, tigecycline, and trimethoprim (27). Incorporating *ramAp* into resistance predictions significantly increased the proportion of isolates predicted to be resistant, particularly for azithromycin (4.1%–13.2%), nalidixic acid (7.9%–17.0%), and tigecycline (0%–9.3%). In contrast, only minor increases were observed for chloramphenicol (from 53.0% to 53.3%), ciprofloxacin nonsusceptibility (from 46.7% to 47.7%), sulfamethoxazole (from 60.9% to 61.3%), tetracycline (from 60.7% to 60.9%), and trimethoprim (from 45.2% to 45.4%). Among *ramAp*-positive isolates, resistance to chloramphenicol, ciprofloxacin, sulfamethoxazole, tetracycline, and trimethoprim was already largely attributable to other established AMR determinants. In contrast, known AMR determinants for azithromycin, nalidixic acid, and tigecycline were relatively uncommon. As a result, the contribution of *ramAp*-associated MIC elevation was more evident for these antimicrobials.

Although several antimicrobials were not included in the AST panel, their resistance rates could be predicted based on AMR determinants identified from WGS data. These antimicrobials included streptomycin (60.1%), kanamycin (22.3%), clindamycin (21.6%), fosfomycin (13.6%), rifampin (11.1%), erythromycin (6.2%), and hygromycin B (2.1%) (Table 1).

### Genotype–phenotype discordance and contributing factors

Genotype–phenotype resistance discordance among the 1,490 *Salmonella* isolates (Table 2) could be categorized into four major types: (i) breakpoint-dependent classification, (ii) reduced or absent phenotypic expression of resistance genes, (iii) MIC modulation by *ramAp*, and (iv) absence of known AMR determinants.

**TABLE 2:** Categories of genotype–phenotype discordance and contributing factors affecting antimicrobial susceptibility among 1,490 *Salmonella* isolates

Category	Antimicrobial	No. isolates	Description of discrepancy	Reference
Breakpoint-dependent classification	Cefotaxime	31	Isolates carrying *bla*_DHA-1_ and *bla*_CTX-M-65_ classified as susceptible or intermediate under CLSI criteria	Table S2_FOT
	Ceftazidime	63	Isolates carrying *ampC*, *bla*_CMY-2_*, bla*_CTX-M-3_*, bla*_CTX-M-65_*,* and *bla*_DHA-1_ classified as susceptible or intermediate under CLSI criteria	Table S2_TAZ
Reduced or absent phenotypic expression of resistance genes	Ampicillin	3	Isolates carrying *bla*_CTX-M_ and *bla*_TEM-1_ exhibited ampicillin MICs within the intermediate range	Table S2_AMP
	Ciprofloxacin	4	Isolates carrying *qnrS1* only exhibited a ciprofloxacin MIC of 0.06 mg/L, below the resistance breakpoint	Table S2_CIP
	Sulfamethoxazole	1	Isolate carrying *sul1* exhibited a sulfamethoxazole MIC of 16 mg/L, below the resistance breakpoint	Table S2_SUL
	Tetracycline	5	Isolates carrying *tet(M*) only exhibited tetracycline MICs of 2–4 mg/L, below the resistance breakpoint	Table S2_TET
	Trimethoprim	1	Isolate carrying *dfrA51* exhibited a trimethoprim MIC of 0.25 mg/L, below the resistance breakpoint	Table S2_TMP
MIC modulation by *ramAp*	Azithromycin	5	Isolates carrying *ramAp* only exhibited elevated MICs reaching the resistance breakpoint	Table S2_AZI
	Chloramphenicol	4	Isolates carrying *ramAp* only exhibited elevated MICs reaching the resistance breakpoint	Table S2_CHL
	Nalidixic acid	100	Isolates carrying *ramAp* only exhibited elevated MICs reaching the resistance breakpoint	Table S2_NAL
	Tigecycline	134	Isolates carrying *ramAp* only exhibited elevated MICs reaching the resistance breakpoint	Table S2_TGE1
Absence of known AMR determinants	Nalidixic acid	3	Isolates lacking known resistance determinants exhibited a nalidixic acid MIC at the resistance breakpoint (32 mg/L)	Table S2_NAL
	Tigecycline	165	Isolates lacking known tigecycline resistance determinants exhibited tigecycline MICs at or above the resistance breakpoint	Table S2_TGE1
	Colistin	91	*S*. Enteritidis isolates lacking known colistin resistance determinants exhibited phenotypic resistance (*N* = 91)	Table S2_COL

The first category comprised breakpoint-dependent classification for cefotaxime and ceftazidime. Using CLSI interpretive criteria for cefotaxime (S ≤ 1, I = 2, R ≥ 4 mg/L), 31 isolates carrying *bla*_CTX-M-65_ or *bla*_DHA-1_ were classified as susceptible or intermediate (Table S2_FOT). Similarly, 63 isolates carrying β-lactamase genes, including *ampC*, *bla*_CMY-2_, *bla*_CTX-M-3_, *bla*_CTX-M-65_, and *bla*_DHA-1_, remained classified as susceptible or intermediate according to the CLSI ceftazidime breakpoints (S ≤ 4, I = 8, R ≥ 16 mg/L) (Table S2_TAZ).

The second category, reduced or absent phenotypic expression of resistance genes, was observed for ampicillin, ciprofloxacin, sulfamethoxazole, tetracycline, and trimethoprim. In these cases, isolates carrying known AMR determinants, including *bla*_CTX-M_, *bla*_TEM-1_, *qnrS1*, *sul1*, *tet(M*), and *dfrA51*, were phenotypically classified as susceptible or intermediate, showing that some isolates carrying these determinants exhibited MICs below the clinical resistance breakpoints (Table S2_AMP, S2_CIP, S2_SUL, S2_TET, and S2_TMP).

The third category involved efflux-associated MIC elevation linked to *ramAp*. For azithromycin and chloramphenicol, five and four isolates carrying *ramAp* alone exhibited MICs at or above the resistance breakpoint (Table S2_AZI and S2_CHL). A similar pattern was observed for nalidixic acid, in which 100 isolates carrying *ramAp* alone exhibited MICs reaching the resistance breakpoint (Table S2_NAL). For tigecycline, 134 isolates carrying *ramAp* alone exhibited MICs at or above the EUCAST resistance breakpoint (Table S2_TGE1).

The fourth category involved phenotypic resistance in the absence of known AMR determinants. For nalidixic acid, three isolates lacking known AMR determinants exhibited MICs at the resistance breakpoint (32 mg/L) (Table S2_NAL). Similarly, 165 isolates lacking known tigecycline resistance determinants nevertheless exhibited tigecycline MICs at or above the EUCAST resistance breakpoint (Table S2_TGE1). For colistin, 91 *S.* Enteritidis isolates lacking known AMR determinants were phenotypically resistant (Table S2_COL).

### Serovar distribution and associated antimicrobial resistance patterns

A total of 61 serovars were identified among 1,490 isolates, with *S*. I 1,4,[5],12:i:- (26.0%) being the most prevalent, followed by *S*. Enteritidis (17.3%) and *S*. Agona (10.1%) (Table 3). Other commonly detected serovars included *S*. Anatum (6.1%), *S*. Newport (4.4%), *S*. Typhimurium (4.4%), *S*. Kentucky (4.2%), *S*. Goldcoast (2.8%), *S*. Stanley (2.8%), and *S*. Infantis (1.9%).

**TABLE 3:** Distribution of *Salmonella* serovars and antimicrobial resistance profiles among 1,490 isolates collected in Taiwan, 2025^*a*^

Serovar	No. isolates	Proportion, %	PS (%)	MDR (%)	XDR (%)	AziR-XDR (%)	MDR clone (%)
I 1,4,[5],12:i:-	387	26.0	5 (1.3)	145 (37.5)	23 (5.9)	17 (4.4)	14 (3.6)
Enteritidis	258	17.3	175 (67.8)	27 (10.5)	0 (0)	0 (0)	0 (0)
Agona	151	10.1	0 (0)	106 (70.2)	90 (59.6)	85 (56.3)	127 (84.1)
Anatum	91	6.1	6 (6.6)	81 (89.0)	81 (89.0)	0 (0)	83 (91.2)
Newport	66	4.4	23 (34.8)	7 (10.6)	0 (0)	0 (0)	0 (0)
Typhimurium	66	4.4	38 (57.6)	19 (28.8)	4 (6.1)	4 (6.1)	0 (0)
Kentucky	62	4.2	0 (0)	58 (93.5)	41 (66.1)	28 (45.2)	60 (96.8)
Goldcoast	41	2.8	1 (2.4)	38 (92.7)	36 (87.8)	32 (78.0)	40 (97.6)
Stanley	41	2.8	39 (95.1)	0 (0)	0 (0)	0 (0)	0 (0)
Infantis	29	1.9	4 (13.8)	25 (86.2)	25 (86.2)	0 (0)	25 (86.2)
Livingstone	27	1.8	6 (22.2)	18 (66.7)	0 (0)	0 (0)	0 (0)
Havana	27	1.8	0 (0)	22 (81.5)	0 (0)	0 (0)	0 (0)
Derby	23	1.5	0 (0)	4 (17.4)	0 (0)	0 (0)	0 (0)
Chester	16	1.1	15 (93.8)	0 (0)	0 (0)	0 (0)	0 (0)
Rissen	16	1.1	3 (18.8)	2 (12.5)	0 (0)	0 (0)	0 (0)
Other 46 serovars	189	12.7	150 (79.4)	21 (11.1)	2 (1.1)	1 (0.5)	1 (0.5)
**Total**	**1,490**	**100**	**465** (**31.2**)	**573** (**38.5**)	**302** (**20.3**)	**167** (**11.2**)	**350** (**23.5**)

^*a*^ PS, pansusceptible (no antimicrobial resistance determinants detected by WGS); MDR, WGS-inferred resistance to ampicillin, chloramphenicol, and trimethoprim–sulfamethoxazole; XDR, MDR with additional nonsusceptibility to fluoroquinolones and resistance to third-generation cephalosporins; AziR-XDR, XDR with additional resistance to azithromycin. MDR clones refer to previously identified high-risk clones in Taiwan, including ICE_erm42-carrying S. Albany (first emerged in 2014), MDR S. Anatum (2015), AziR-XDR S. Typhimurium (2016), MDR S. Goldcoast (2017), MDR S. Agona (2018), MDR S. Infantis (2021), and AziR-XDR S. Kentucky (2024). Percentages represent the proportions of MDR clone isolates within each serovar.

Antimicrobial resistance profiles predicted by WGS varied markedly across serovars. Among the 15 most common serovars, a high proportion of pansusceptible isolates was observed in *S*. Stanley (95.1%), *S*. Chester (93.8%), *S*. Enteritidis (67.8%), and *S*. Typhimurium (57.6%). In contrast, MDR was highly prevalent in *S*. Kentucky (93.5%), *S*. Goldcoast (92.7%), *S*. Anatum (89.0%), *S*. Infantis (86.2%), *S*. Havana (81.5%), and *S*. Agona (70.2%). XDR phenotypes were most frequently observed in *S*. Anatum (89.0%), *S*. Goldcoast (87.8%), *S*. Infantis (86.2%), *S*. Kentucky (66.1%), and *S*. Agona (59.6%). AziR-XDR isolates were predominantly identified in *S*. Goldcoast (78.0%), *S*. Agona (56.3%), *S*. Kentucky (45.2%), with lower proportions observed in *S*. Typhimurium (6.1%), and *S*. I 1,4,[5],12:i:- (4.4%). Overall, 31.2% of isolates were pansusceptible, 38.5% were MDR, 20.3% were XDR, and 11.2% were AziR-XDR.

### Epidemiological distribution and persistence of major MDR clones

Previously identified MDR clones from long-term surveillance (2004–2024), including ICE_erm42-carrying *S*. Albany (28), MDR *S*. Anatum (6), *S*. Goldcoast (5), *S*. Agona (8), *S*. Infantis (9), and *S*. Typhimurium (7), as well as MDR *S*. Kentucky newly identified in 2025 (10), were detected in the 2025 isolate collection. Based on WGS-derived phylogenetic analysis and resistance profiles, these MDR clones constituted high proportions within their respective serovars, including *S*. Goldcoast (97.6%), *S*. Kentucky (96.8%), *S*. Anatum (91.2%), *S*. Infantis (86.2%), and *S*. Agona (84.1%). One ICE_erm42-carrying *S*. Albany was also detected in this isolate collection. In total, these MDR clones accounted for 23.5% of all isolates (Table 3).

#### Association of *ramAp* with MIC elevation

In this study, the effects of the plasmid-borne *ramAp* were evaluated on azithromycin, nalidixic acid, and tigecycline, as sufficient isolates carrying *ramAp* alone were available for analysis. MIC distributions revealed consistent shifts toward higher MIC values for isolates carrying *ramAp* alone compared to wild-type isolates. For azithromycin and tigecycline, MIC distributions shifted by approximately 4-fold, while nalidixic acid exhibited a more pronounced shift of ≥8-fold (Fig. S2 to S4).

Thirty-six *S*. Enteritidis isolates carrying *ramAp* were excluded from the antimicrobial mapping analysis because they lacked an upstream promoter region, indicating that *ramAp* was unlikely to be expressed in these isolates.

## DISCUSSION

This study provides a comprehensive evaluation of genotype–phenotype resistance in 1,490 *Salmonella* isolates using ONT-based WGS data in a nationwide surveillance setting. Overall, genotypic resistance predictions demonstrated high concordance with phenotypic AST results across most antimicrobial classes, supporting the utility of ONT-WGS as a practical tool for large-scale AMR identification and surveillance (Table 1). Concordance was particularly high for several clinically important antimicrobials, including ampicillin, azithromycin, chloramphenicol, ciprofloxacin, gentamicin, meropenem, sulfamethoxazole, tetracycline, and trimethoprim, for which phenotypic and genotypic resistance rates differed by ≤0.5% (Table 1). In addition to predicting resistance phenotypes, WGS data also provided detailed information on AMR determinants, resistance-associated mutations, and the genetic contexts of resistance genes that could not be obtained from conventional AST alone. However, notable genotype–phenotype discrepancies remained for selected antimicrobial classes, particularly third-generation cephalosporins, tigecycline, and colistin. These discordances were not random but were associated with several biologically and methodologically distinct mechanisms, including breakpoint-dependent classification, reduced or absent phenotypic expression of resistance genes, MIC modulation by *ramAp*, and the absence of known AMR determinants (Table 2).

Breakpoint-dependent classification contributed to discordance for β-lactam antimicrobials, particularly cefotaxime and ceftazidime. Ninety-four isolates carrying β-lactamase genes, including *bla*_CTX-M-65_ and *bla*_DHA-1_, were classified as susceptible or intermediate according to CLSI criteria. This discordance was most evident for cefotaxime, where among 117 *bla*_DHA-1_-carrying isolates, 30 (25.6%) exhibited MICs of 1–2 mg/L and therefore fell below the resistance breakpoint (Table S2_FOT). A similar pattern was observed for ceftazidime, where 25 of 26 *bla*_CTX-M-65_-carrying isolates exhibited MICs of 2–4 mg/L and were categorized as susceptible (Table S2_TAZ). In addition, 33 (28.2%) of *bla*_DHA-1_-carrying isolates were classified as non-resistant. These findings suggest that current CLSI clinical breakpoints may not always distinguish wild-type isolates from isolates harboring clinically important β-lactamase genes. This apparent genotype–phenotype discordance largely reflects the limitations of current clinical breakpoint interpretation rather than the absence of clinically relevant resistance mechanisms. While CLSI clinical breakpoints are primarily intended to guide antimicrobial therapy, EUCAST epidemiological cutoff values (ECOFFs) are designed to identify isolates harboring acquired resistance mechanisms. Therefore, integrating WGS-derived ESBL gene detection with conventional AST may improve both clinical interpretation and public health surveillance, particularly for isolates with MICs near current clinical breakpoints.

Importantly, such discordance has been associated with adverse clinical outcomes. Previous studies have demonstrated that infections caused by ESBL-producing organisms may result in treatment failure when treated with cephalosporins despite *in vitro* susceptibility. In a landmark study, Paterson et al. reported failure rates of 54% among patients infected with ESBL-producing organisms classified as susceptible and 100% among those with intermediate MICs (29). Pharmacokinetic and pharmacodynamic analyses further support these observations, showing that as MIC values approach clinical breakpoints, the probability of achieving pharmacodynamic targets (e.g., %T > MIC) declines substantially, even for isolates categorized as susceptible, thereby compromising therapeutic efficacy (30). Our data highlight that the current interpretive criteria (clinical breakpoints) for cefotaxime and ceftazidime do not effectively distinguish wild-type from non-wild-type populations. These isolates may be misclassified as non-resistant despite carrying AMR determinants, increasing the risk of treatment failure. In contrast, epidemiological cutoff values (ECOFFs) provide a biologically meaningful framework for distinguishing wild-type from non-wild-type populations, thereby improving concordance between genotypic AMR predictions and phenotypic susceptibility results. In our data, the MIC distributions suggest tentative ECOFFs of >0.5 mg/L (≥1 mg/L) for cefotaxime (Table S2_FOT) and >1 mg/L (≥2 mg/L) for ceftazidime (Table S2_TAZ), which more effectively separate wild-type isolates from those carrying β-lactamase determinants and reduce the risk of misclassification.

The plasmid-borne regulator *ramAp* plays a crucial role in complicating the interpretation of genotype–phenotype resistance. Unlike conventional resistance genes that usually confer high-level resistance, *ramAp* increases MICs by activating multidrug efflux systems, thereby reducing intracellular antimicrobial concentrations (27). Consistent with this mechanism, *ramAp* was associated with approximately fourfold increases in MICs for azithromycin (Fig. S2) and tigecycline (Fig. S4) and at least eightfold increases for nalidixic acid (Fig. S3). Despite these marked MIC shifts, most *ramAp*-carrying isolates remained below current clinical breakpoints and were therefore classified as susceptible or intermediate. Consequently, isolates with reduced susceptibility may still be interpreted as clinically susceptible despite harboring a genomic determinant associated with elevated MICs. From a clinical perspective, such MIC shifts may narrow pharmacodynamic margins and potentially compromise treatment efficacy, particularly when MICs approach clinical breakpoints. The present study substantially extends our previous mechanistic characterization of *ramAp* by validating its effects on antimicrobial susceptibility in a large nationwide surveillance data set. The consistent MIC shifts observed across genetically diverse *Salmonella* isolates support the inclusion of *ramAp* in WGS-based AMR interpretation, as it serves as a robust genomic marker of reduced antimicrobial susceptibility.

Substantial genotype–phenotype discordance was observed for tigecycline and colistin (Table 1). For tigecycline, 20.1% of isolates were classified as phenotypically resistant according to the current EUCAST clinical breakpoint, yet no known high-level resistance determinants, such as *tet(X*), were detected (31, 32). Because the current EUCAST breakpoint for tigecycline is relatively low, much of the observed discordance likely reflects low-level MIC elevations around the clinical breakpoint rather than unequivocal high-level resistance. Consistent with this interpretation, 134 of the 299 tigecycline-resistant isolates carried *ramAp*, which was associated with an approximately fourfold increase in tigecycline MICs (Fig. S4), indicating that *ramAp* primarily contributes to MIC elevation rather than conferring high-level resistance. In contrast, the remaining 165 isolates lacked both *ramAp* and any currently recognized tigecycline resistance determinants, suggesting that additional mechanisms contributing to modest increases in MICs remain to be identified (Table S2_TGE2). Previous studies have suggested that specific Tet(A) variants or elevated tet(A) expression may contribute to reduced tigecycline susceptibility (33, 34); however, currently available WGS-based bioinformatic approaches do not reliably distinguish these functional variants or their expression status. Collectively, these findings suggest that accurate genome-based prediction of tigecycline resistance remains challenging.

The most striking genotype–phenotype discordance was observed for colistin in *S.* Enteritidis. A high prevalence of phenotypic resistance was observed, with 35.7% (92/258) of isolates exceeding the clinical breakpoint, yet only one isolate carried a known AMR determinant (*mcr-1.1*). Among isolates lacking known colistin resistance determinants, 35.4% (91/257) exhibited MICs at or above the resistance threshold, accompanied by a right-shifted MIC distribution (Fig. S1). This pattern is consistent with previous reports from Taiwan and China, where elevated colistin MICs in *S*. Enteritidis were not explained *by mcr* genes *or canonical chromosomal mutations* (4, 35). Notably, mutations in known regulatory systems (e.g., pmrAB and phoPQ) are rarely detected, suggesting that currently recognized resistance mechanisms cannot fully explain this phenotype. Previous studies by Agersø et al. demonstrated that reduced colistin susceptibility is also observed in *S. Dublin*, another O:1,9,12 serovar (36), while Ricci et al. further proposed that O-antigen composition is a major determinant of colistin susceptibility (37). Collectively, these observations suggest that the elevated colistin MICs observed in *S.* Enteritidis may reflect a serogroup-associated intrinsic reduced susceptibility rather than a conventional acquired resistance mechanism. Although differences in O-antigen structure have been proposed to contribute to the reduced susceptibility of O:1,9,12 serovars, the precise molecular basis remains unresolved and requires further investigation.

Another category of discordance was reduced or absent phenotypic expression of resistance genes, although such cases were infrequent in this study (Table 2). Specifically, five isolates carrying *tet(M*) exhibited low tetracycline MICs (2–4 mg/L) and were phenotypically susceptible despite the absence of obvious disruptions in the coding sequence and upstream promoter region (Table S2_TET). Similarly, isolates carrying other resistance genes, such as *qnrS1* for ciprofloxacin and *sul1* for sulfamethoxazole, showed low MICs below resistance breakpoints (Table S2_CIP and Table S2_SUL). These observations indicate that the presence of these ARGs alone is insufficient to predict phenotypic resistance. Additional biological factors, including transcriptional regulation, protein functionality, and other post-genomic mechanisms, may determine whether these genes confer phenotypic resistance.

In addition, some discordant cases were attributed to gene truncation or regulatory disruption. For example, nine *cmlA1*-carrying *S.* Rissen isolates, initially annotated as having complete genes based on the default coverage threshold (≥90%), were later found to have partial sequences (coverage 90.7%) upon re-analysis due to genotype–phenotype inconsistency. Similarly, *aac(3)-IVa* in two isolates exhibited an IS26-mediated promoter interruption, explaining the absence of the gentamicin resistance phenotype. These findings underscore the importance of considering both gene integrity and regulatory context in genomic analyses.

In addition to AMR profiling, this study underscores the value of WGS-based surveillance in monitoring the epidemiological dynamics of previously identified high-risk MDR clones in Taiwan. As shown in Table 3, serovars associated with these MDR clones, including *S.* Anatum (first identified in 2015), *S.* Goldcoast (2017), *S.* Agona (2018), *S.* Infantis (2021), and *S.* Kentucky (2024), remained among the most prevalent serovars in 2025. Notably, MDR clones continued to represent the dominant proportion within their respective serovars, comprising 91.2% of *S.* Anatum, 97.6% of *S.* Goldcoast, 84.1% of *S.* Agona, 86.2% of *S.* Infantis, and 96.8% of *S.* Kentucky. These findings indicate the ongoing circulation and persistence of established MDR clones over time, rather than the sporadic or transient emergence of new clones. The high proportion of MDR clones within these serovars suggests that clonal expansion remains a key driver of antimicrobial resistance in *Salmonella* populations in Taiwan. Collectively, these results highlight the importance of integrating WGS into routine surveillance, not only for resistance prediction but also for tracking the long-term persistence and spread of high-risk clones.

In conclusion, this study demonstrates that ONT-WGS provides a highly reliable method for AMR prediction in *Salmonella*, with genotype–phenotype concordance observed in the vast majority of isolates. However, genotype–phenotype discordance remained for selected antimicrobials, particularly tigecycline and colistin, highlighting areas where current genome-based AMR prediction requires further refinement. Despite these exceptions, ONT-WGS provides faster and more comprehensive AMR data than conventional AST and could complement, and in some applications potentially replace, conventional AST, thereby supporting both clinical decision-making and epidemiological surveillance. Its short turnaround time and flexible sequencing capacity make it readily applicable across laboratories with varying workloads, supporting its broader implementation as a practical platform for routine genomic AMR surveillance.

## Data Availability

The whole-genome sequencing data generated in this study have been deposited in the NCBI database under BioProject PRJNA478278. BioSample and SRA accession numbers for the 1,490 isolates are listed in Table S1.

## SUPPLEMENTAL MATERIAL

Supplemental figures spectrum.01797-26-s0001.pdf Fig. S1 to S4.

Supplemental tables spectrum.01797-26-s0002.xlsx Tables S1 and S2.

ASM does not own the copyrights to Supplemental Material that may be linked to, or accessed through, an article. The authors have granted ASM a non-exclusive, world-wide license to publish the Supplemental Material files. Please contact the corresponding author directly for reuse.
