# Supplementary material for: Evaluation of Oxford nanopore sequencing for antimicrobial resistance surveillance in Salmonella: comparison with phenotypic antimicrobial susceptibility in a large-scale study
Source: Microbiol Spectr. 2026 Aug 12;14(9):e01797-26. doi: 10.1128/spectrum.01797-26 (PMC13532270; doi:10.1128/spectrum.01797-26)
Supplement: Supplementary file 1 [file spectrum.01797-26-s0001.pdf]

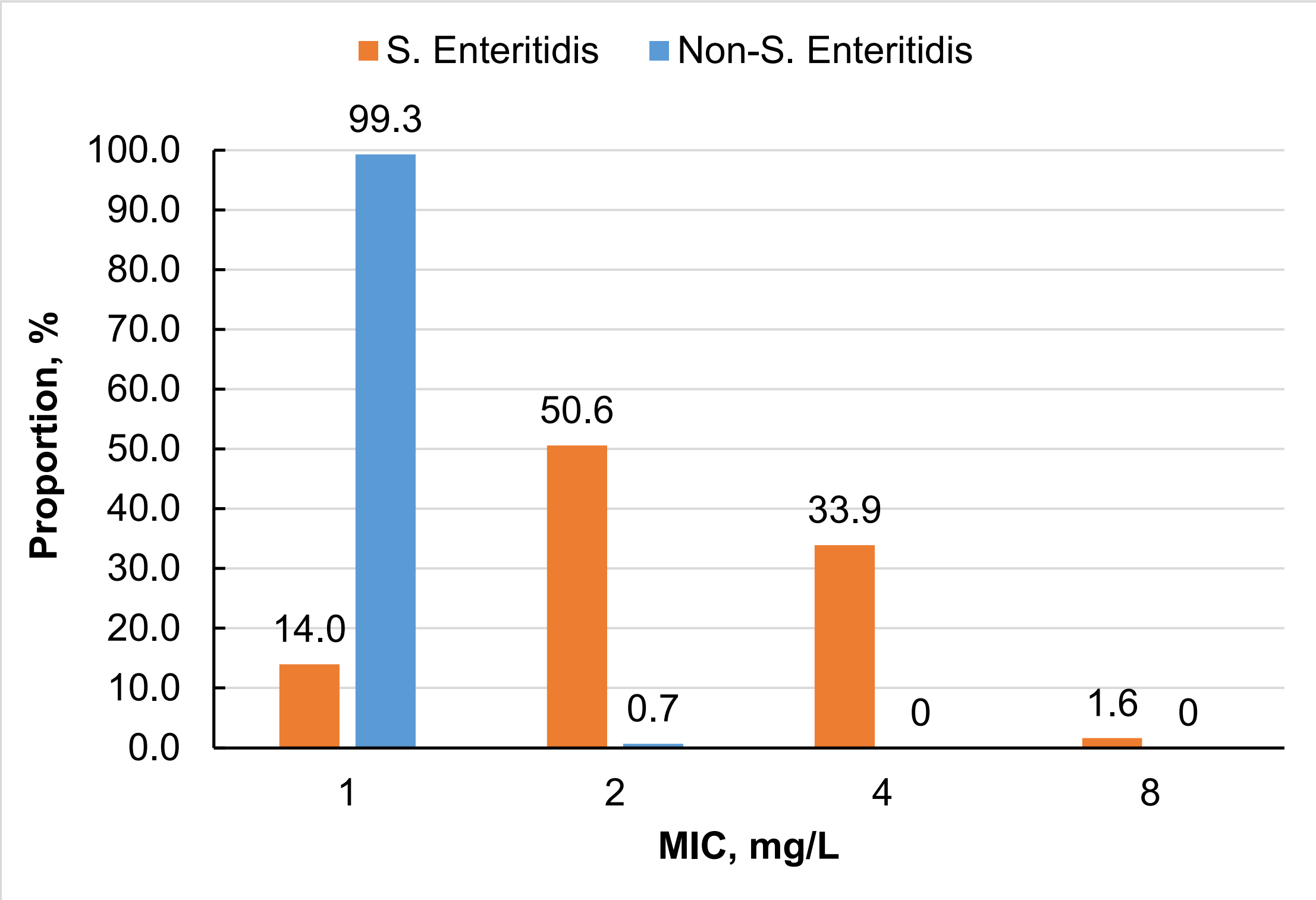

Figure S1. Distribution of colistin minimum inhibitory concentrations (MICs) among *Salmonella* Enteritidis isolates (N = 257) and non-Enteritidis *Salmonella* isolates lacking known colistin resistance determinants (N = 1,223).

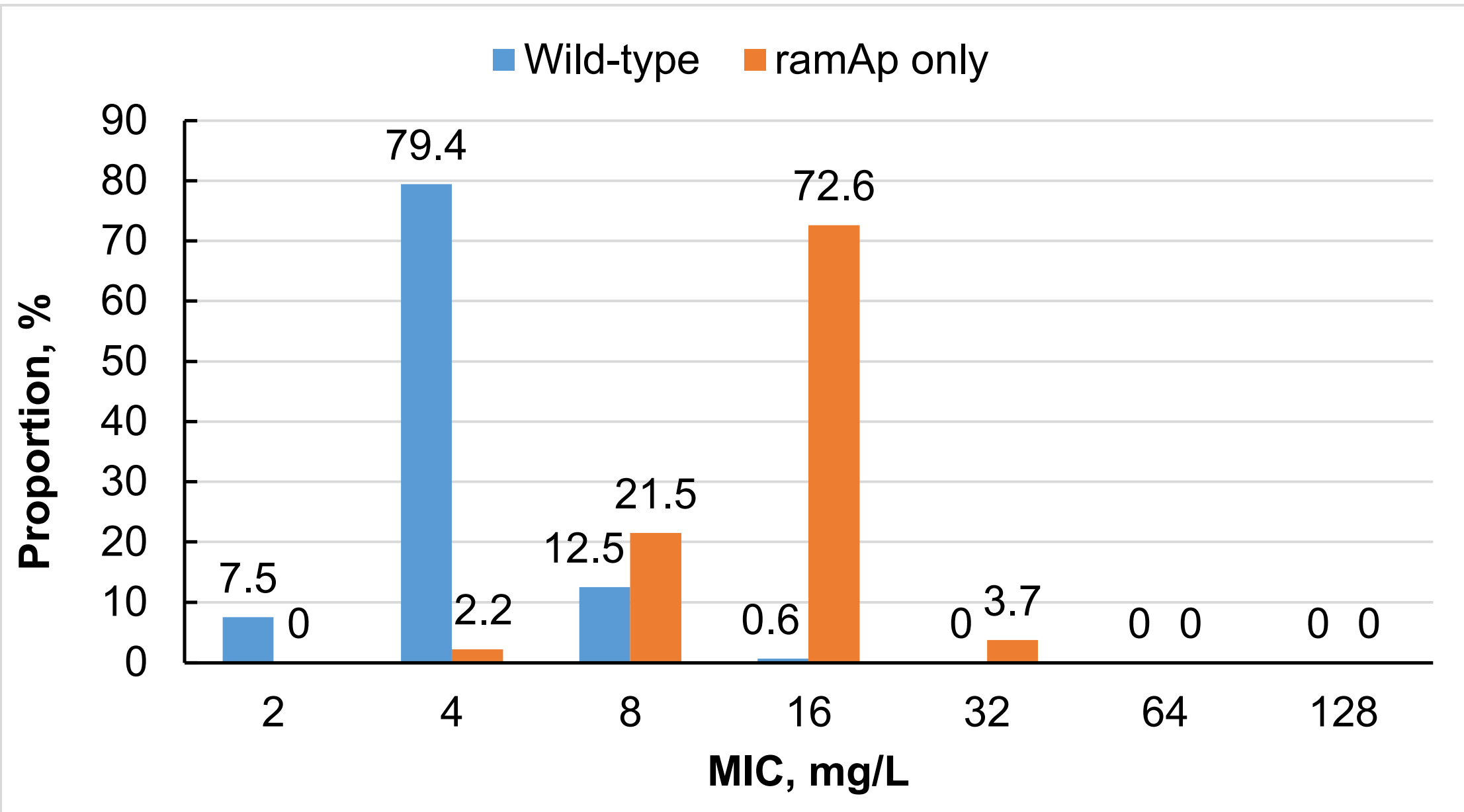

Figure S2. Distribution of azithromycin minimum inhibitory concentrations (MICs) among *Salmonella* isolates lacking known resistance determinants (wild-type) and those carrying *ramAp* alone.

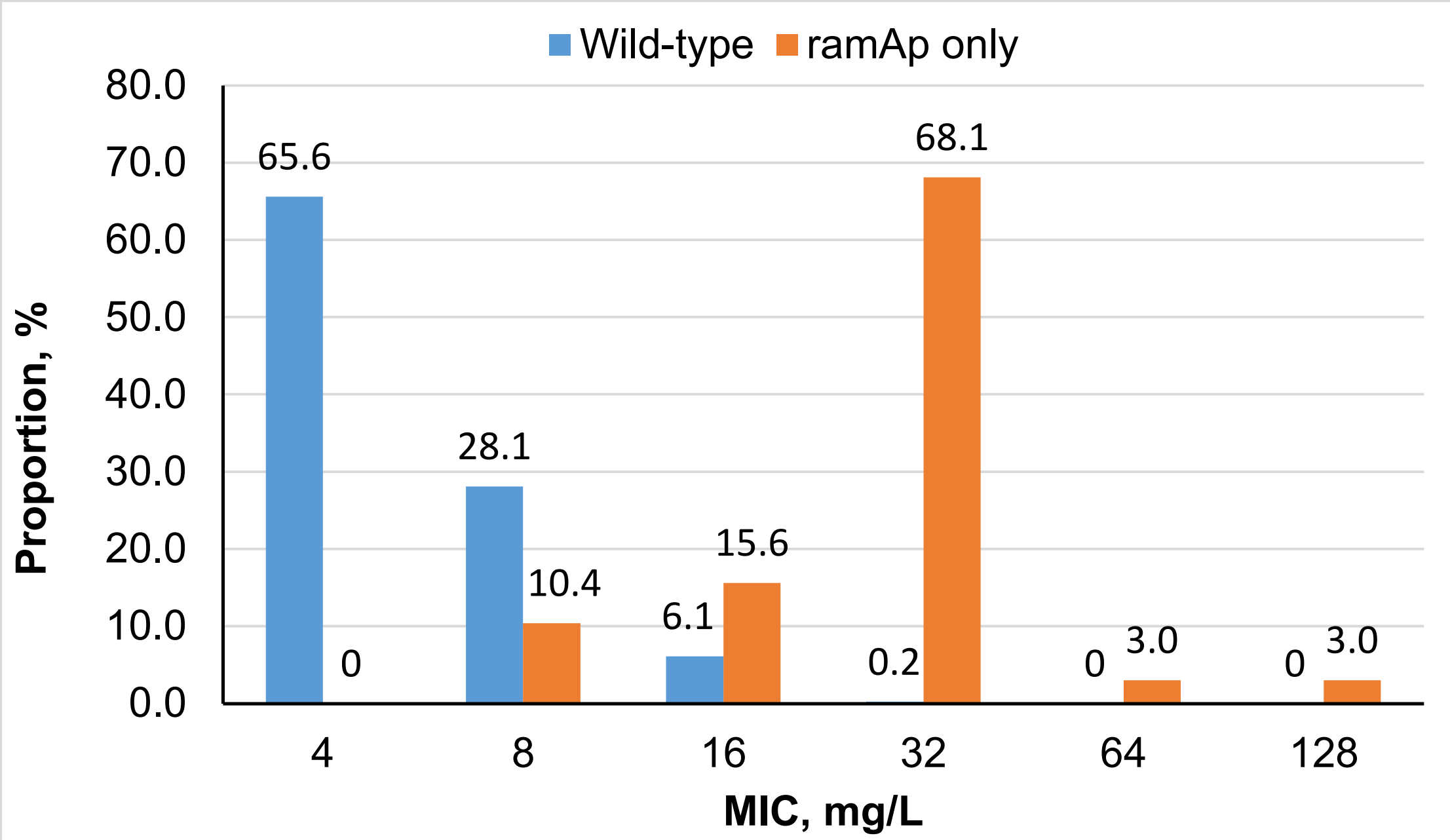

Figure S3. Distribution of nalidixic acid minimum inhibitory concentrations (MICs) among *Salmonella* isolates lacking known resistance determinants (wild-type) and those carrying *ramAp* alone.

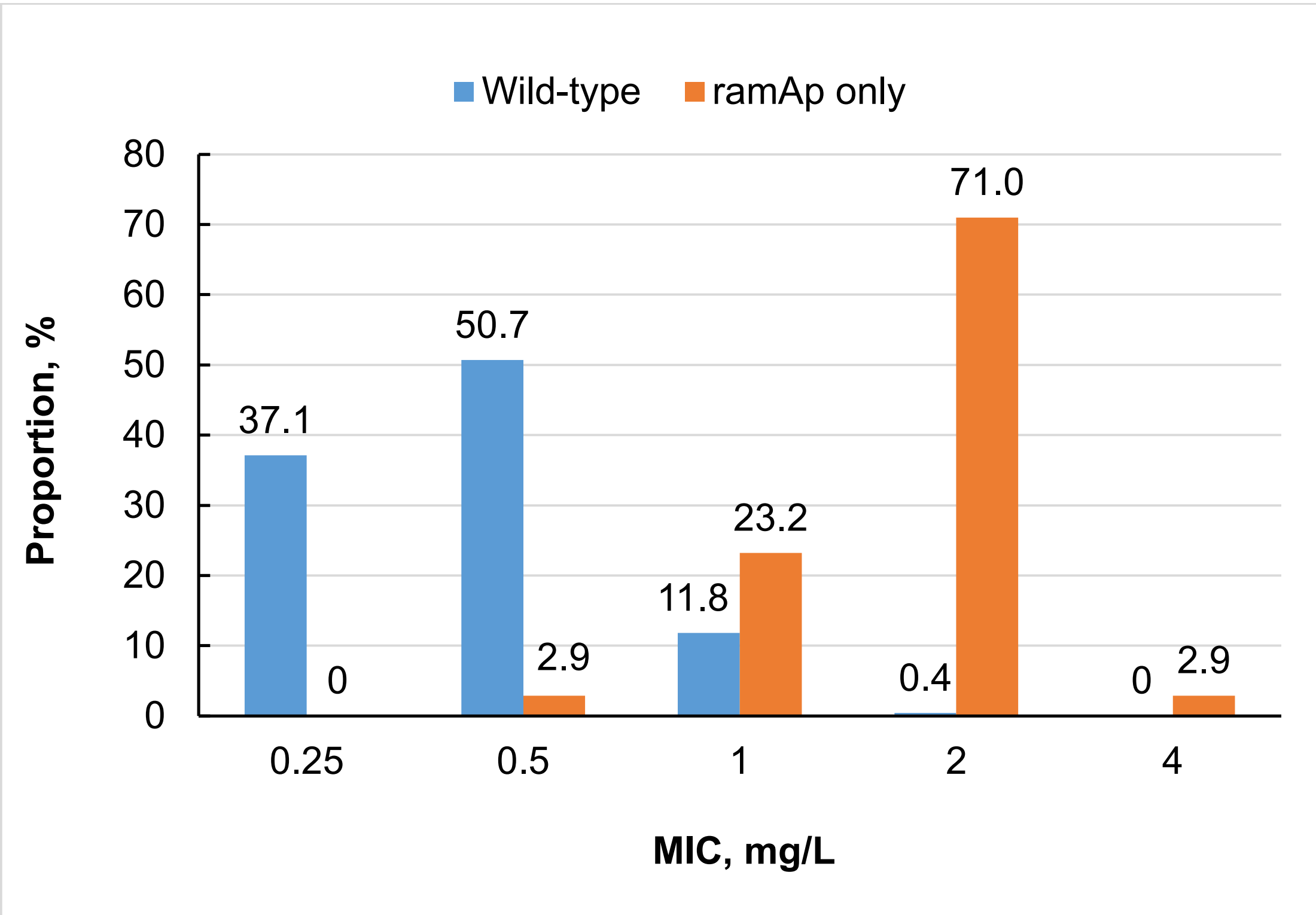

Figure S4. Distribution of tigecycline minimum inhibitory concentrations (MICs) among *Salmonella* isolates lacking known tigecycline resistance determinants (wild-type) and those carrying *ramAp* alone.
